# Opportunistic Coccidiosis in Brazil: Diagnostic Challenges and Epidemiological Gaps in Immunosuppressed Groups

**DOI:** 10.1155/jotm/7297545

**Published:** 2026-08-25

**Authors:** Willian Cardoso Ferreira Zorrer, Estefani Rinaldi, Bibiana Rodrigues de Freitas, Pedro Machado Medeiros de Albuquerque, Natalia Machado Rahal, Rodrigo Casquero Cunha, Fabio Raphael Pascotti Bruhn

**Affiliations:** ^1^ Veterinary Molecular Biology Laboratory, Federal University of Pelotas, Pelotas, Brazil, ufpel.edu.br; ^2^ Zoonosis Control Center, Federal University of Pelotas, Pelotas, Brazil, ufpel.edu.br

**Keywords:** *cryptosporidium*, immunosuppression, public health surveillance, underreporting

## Abstract

Opportunistic infections caused by the protozoa *Cryptosporidium* spp. and *Cystoisospora belli* represent a challenge for public health, particularly in immunocompromised patients. In Brazil, a country with continental dimensions and socioeconomic diversity, the epidemiology of these intestinal coccidioses is complex and often underestimated. This review article aims to consolidate current knowledge on *Cryptosporidium* and *Cystoisospora* infections in immunocompromised individuals within the Brazilian context, addressing clinical manifestations, epidemiology, risk factors, diagnostic methods, treatment options, prevention and control strategies, and challenges related to underreporting. A thorough understanding of these aspects is crucial for improving health policies, optimizing clinical management, and reducing the morbidity and mortality associated with these parasitic diseases.

## 1. Introduction

Intestinal coccidioses, caused by protozoa of the phylum Apicomplexa, such as *Cryptosporidium* spp. and *Cystoisospora belli* (formerly known as *Isospora belli*), are globally recognized as significant agents of diarrhea, especially in vulnerable populations [1, 2]. In immunocompetent individuals, these infections typically present as acute, self‐limiting diarrheal episodes. However, in patients with compromised immune systems, such as those living with HIV/AIDS, organ transplant recipients, or individuals undergoing immunosuppressive therapy or chemotherapy, these parasitic infections can lead to severe, chronic, and potentially fatal conditions, characterized by severe dehydration, intestinal malabsorption, and progressive malnutrition [1, 3, 4].

In Brazil, the occurrence of these infections is influenced by a complex interplay of socioeconomic, environmental, and healthcare‐related factors. Poor sanitation infrastructure in many regions, contamination of water and food, and proximity to animal reservoirs contribute to the spread of infectious oocysts [5]. Despite their clinical relevance, the true burden of cryptosporidiosis and cystoisosporiasis in immunosuppressed patients in Brazil remains poorly understood, partly due to diagnostic gaps that lead to underreporting.

This article reviews the scientific literature on *Cryptosporidium* spp. and *Cystoisospora belli* infections in immunosuppressed patients in Brazil, focusing on clinical presentation, epidemiology, risk factors, and challenges in underreporting, aiming to provide an updated epidemiological overview for public health.

## 2. Search Strategy

This is a narrative, nonsystematic review of the occurrence of *Cryptosporidium* spp. and *Cystoisospora belli* in immunosuppressed patients in Brazil; it does not follow PRISMA reporting, but the search process is described here in full for transparency.

The primary search was conducted in PubMed and Google Scholar, executed on the search strings (“*Cryptosporidium*” OR “*Cystoisospora belli*” OR “*Isospora belli*”) AND (“Brazil” OR “Brasil”) AND (“immunosuppress” OR “HIV” OR “AIDS” OR “transplant” OR “chemotherapy” OR “cancer”) AND (“diagnosis” OR “epidemiology” OR “underreporting” OR “prevalence”), combining equivalent Portuguese‐language terms in a parallel search string. Boolean operators (AND/OR) and truncation were applied as shown. No language filter was applied at the database level, but retrieved records were screened for English‐ or Portuguese‐language full text. This revision additionally incorporated two supplementary, nonsystematic searches conducted by the author team using the same terms: a search of SciELO Brazil and a search of LILACS (via the BVS portal), both of which identified further Brazilian primary studies not indexed in the initial PubMed/Google Scholar search (detailed in the Epidemiology section). Separately, the corresponding author conducted a manual search of Scopus using equivalent terms and supplied the resulting records for evaluation; three of these were assessed as relevant and have been incorporated (detailed in the Epidemiology section), while a fourth, concerning a different parasite outside this review’s scope, was excluded. Web of Science and Embase were not searched in this review, as the author team does not currently have institutional access to either platform; this is acknowledged as a limitation of the present search.

Records were screened independently in duplicate, with disagreements resolved by discussion among the author team; duplicates across databases were removed manually by title/DOI comparison. Eligible records were original articles, case reports, and reviews published between 2000 and 2025 focusing on epidemiological, clinical, or diagnostic findings on cryptosporidiosis or cystoisosporiasis specifically in immunosuppressed populations within Brazilian territory. Studies not focused on immunosuppressed populations, or conducted entirely outside Brazil, were excluded. For studies retained, the following data were extracted where available: publication year, geographic region, patient population, sample size, diagnostic method, reported prevalence, and study design. It should be highlighted that, although the use of the most recent bibliography was prioritized, the status of these protozoa as neglected pathogens results in a relative scarcity of updated primary research in Brazil, which limits the availability of recent, high‐quality data, a constraint that is itself one of the findings of this review rather than only a methodological caveat. As this is a narrative review of published data, no ethical approval was required.

## 3. Etiological Agents and Biological Cycle

The genus *Cryptosporidium* comprises several species, with *C. hominis* and *C. parvum* being the most frequently associated with human infections. Transmission occurs through ingestion of sporulated oocysts excreted in the feces of infected hosts, contaminating water, food, or via direct person‐to‐person or animal‐to‐person contact [1, 3]. The oocysts are highly resistant to environmental conditions and common disinfectants, such as chlorine.

*Cystoisospora belli* is a monoxenous protozoan, with humans as its only known host. Infection occurs through ingestion of mature oocysts present in water or food contaminated with human feces. The oocysts are excreted immature and require time in the environment to sporulate and become infectious [2].

In both cases, after ingestion, sporozoites are released in the small intestine, invade enterocytes, and initiate cycles of asexual and sexual reproduction, culminating in the formation of new oocysts that are excreted in feces, perpetuating the cycle. In immunosuppressed individuals, replication can be continuous and intense, leading to chronic infection.

## 4. Epidemiology in Brazil Among Immunosuppressed Patients

The epidemiology of cryptosporidiosis and cystoisosporiasis in immunosuppressed patients in Brazil is heterogeneous and unevenly studied. Most available data come from patients living with HIV/AIDS, who constitute the most‐studied risk group; other immunosuppressed groups remain comparatively neglected in the primary literature, as discussed below.

### 4.1. Prevalence and Geographic Distribution

Studies conducted in different regions of Brazil show significant variations in the prevalence of these infections, and much of that variation tracks the diagnostic method used rather than true underlying differences. In HIV/AIDS patients, the prevalence of *Cryptosporidium* spp. can vary considerably. A study in Ribeirão Preto (SP) found a frequency of 6.4% for *Cryptosporidium* sp. and 4.4% for *C. belli* in HIV‐positive patients [6].

A recent systematic review compiling 48 human studies in Brazil indicates that *Cryptosporidium* infection is widespread, although research remains heavily concentrated in the Southeast region, particularly São Paulo [7]. This geographical concentration is itself a research artifact, not only an epidemiological one: it likely reflects where reference laboratories, academic parasitology groups, and molecular diagnostic infrastructure are physically located, meaning apparent regional differences in prevalence partly measure laboratory capacity rather than true differences in transmission. This leaves significant epidemiological gaps in the North and Central‐West regions, resulting in an incomplete national profile [7]. A supplementary LILACS search for this revision identified one relevant older study from the North region—a 1989 investigation of cryptosporidiosis in children with acute diarrhea in Belém, Pará, reporting a 5.2% positivity—but this concerns a general pediatric population, not an immunosuppressed one. We must also correct a claim made in the previous revision: we had stated that no primary data existed for immunosuppressed populations in the Central‐West region. This was incorrect. A cross‐sectional study of 90 HIV/AIDS patients in Jataí, state of Goiás (Central‐West region), specifically investigated *Cryptosporidium* spp. and *Cystoisospora belli* alongside *Strongyloides stercoralis*, finding low but non‐zero positivity (1.1% and 1.1%, respectively) and documenting significant associations between parasitic infection and clinical AIDS status, ART use, and CD4^+^ count [8]. We correct our earlier statement accordingly: primary data from the Central‐West region does exist, though it remains limited to this single institution and shows markedly lower coccidia positivity than studies from the Southeast—itself worth noting, since it could reflect either true lower regional prevalence, a smaller sample of severely immunosuppressed (AIDS‐stage, low‐CD4) patients specifically or continued underdetection. For the North region, despite this and prior targeted searches, no peer‐reviewed primary study specifically addressing cryptosporidiosis or cystoisosporiasis in immunosuppressed patients was identified; only general pediatric population data (Loureiro et al., Pará) exists there [9].

Furthermore, mean positivity rates vary significantly according to the diagnostic approach, ranging from ∼8.9% using direct methods to ∼52.2% with indirect methods [7]. This roughly sixfold spread is the clearest quantitative illustration in the Brazilian literature that “underreporting” is, in substantial part, a measurement problem: a large share of the apparent gap between reported and true prevalence is attributable to which assay was used, not only to undertesting. This infection is notably frequent among immunocompromised individuals, including people living with HIV and those with renal diseases [7], aligning with One Health perspectives that highlight the increased clinical burden in such vulnerabilities [10].

Another study in Rio Grande do Norte, involving hospitalized HIV/AIDS patients, also investigated the frequency of these coccidia, highlighting the lack of data, particularly in the Northeast region [5]. A more recent study on the prevalence and genetic characterization of *Cryptosporidium* spp. and *Cystoisospora belli* in HIV‐infected patients in Brazil observed a significant association between nonadherence to antiretroviral therapy (ART) and the presence of these parasites [11].

The prevalence of *Cystoisospora belli* in HIV‐positive patients in Brazil also varies, with reported rates ranging from 4.4% to 18% in different studies [12]. Seasonality also appears to influence occurrence, with some studies suggesting higher frequencies of *Cryptosporidium* spp. during rainy months and *C. belli* during warm and humid months, although both microorganisms can remain viable in the environment for months due to their resistance to environmental conditions [6, 11].

Beyond HIV/AIDS patients, other immunosuppressed groups have somewhat better Brazilian primary data than the previous version of this review reflected, though the evidence base remains thin and largely single center. A case–control study compared renal transplant recipients, hemodialysis patients, and nonimmunosuppressed controls, reporting *Cryptosporidium parvum* frequencies of 34.8%, 25%, and 17.4%, respectively—a gradient consistent with immunosuppression intensity, though the difference did not reach statistical significance in that study’s sample size [13]. Two further Brazilian hemodialysis‐specific studies exist: an open controlled study found *Cryptosporidium* and other enteroparasites with pathogenic potential in hemodialysis patients [14], and an earlier study in São Paulo reported intestinal parasitism, including coccidia, among hemodialysis patients alongside associated symptomatology [15]—together, these three studies make hemodialysis/transplant populations the best‐documented non‐HIV immunosuppressed group in the Brazilian literature, though still limited to a handful of institutions. A molecular characterization study of kidney transplant recipients in Rio de Janeiro identified *Cryptosporidium* subtypes associated with greater virulence circulating specifically within this population, the first such report in Brazilian transplant patients [16].

In pediatric HIV‐specific immunosuppression, a cross‐sectional cohort study of 17 HIV‐infected children in Ribeirão Preto (SP) found *Cryptosporidium* spp. in 23.5%—a rate the study’s own authors note is higher than reported *Cryptosporidium* prevalence among healthy Brazilian children (≤ 15.5%) and documented household coinfection with family members in 70% of cases, implicating shared socioenvironmental exposure alongside the child’s immune status [17]. A cross‐sectional study in the Triângulo Mineiro region additionally examined the seasonal profile of cryptosporidiosis and cystoisosporiasis specifically in relation to CD4^+^ lymphocyte levels in HIV/AIDS patients, directly supporting this review’s claim (above) that CD4 count and seasonality both modulate risk [18]. Separately, a case report presented at the Brazilian Congress of Infectious Diseases (published as a congress abstract within a peer‐reviewed journal’s conference supplement, not a full independently peer‐reviewed article) describes disseminated cryptosporidiosis with gastrointestinal and pulmonary involvement in a child undergoing bone marrow transplantation for CD40‐ligand deficiency (Hyper‐IgM syndrome), successfully managed with high‐dose nitazoxanide and azithromycin [19]. We flag its abstract‐only status explicitly since it carries less evidentiary weight than a peer‐reviewed case report; note also that two independent sources report slightly different page/article numbers for this same abstract, which the authors should verify directly against the publisher before citing. For baseline pediatric context (not immunosuppression‐specific), a daycare‐based study in Recife found *Cryptosporidium* positivity in 32.4% of children aged 1–14 years, illustrating how common exposure is even outside clinical immunosuppression and underscoring how much more severe and prolonged the same exposure becomes once cellular immunity is impaired [20] (Table 1).

**TABLE 1:** Epidemiological data of opportunistic coccidiosis in Brazil.

Study/year	Region	Design	Population (n)	Diagnostic method	*Cryptosporidium* spp. Finding	*Cystoisospora belli* finding	Source
Instituto Adolfo Lutz, 2000	Ribeirão Preto (SP), Southeast	Cross‐sectional	HIV/AIDS patients	Microscopy (modified Ziehl–Neelsen)	6.4%	4.4%	Instituto Adolfo Lutz, [6]
Moura et al., 2017	Natal (RN), Northeast	Cross‐sectional	Hospitalized HIV/AIDS patients	Microscopy	Not reported (frequency documented, exact value not extracted)	Not reported	Moura et al., 2017 [5]
Assis et al., 2013	São Paulo, Southeast	Cross‐sectional	HIV‐infected patients	Microscopy + molecular characterization	4.4%–18% range across studies cited	4.4%–18% range across studies cited	Assis et al., 2013 [12]
Renal transplant/hemodialysis case–control study	Not specified in source, Southeast (institutional)	Case–control	Renal transplant (*n* = 23), hemodialysis (*n* = 32), controls (*n* = 27)	Repeated fecal microscopy (1–6 exams/patient)	34.8% (transplant); 25% (hemodialysis); 17.4% (control)	Not applicable (C. parvum only)	Chieffi et al., 1998 [13]
Hemodialysis, open controlled study	Not specified, Southeast (institutional)	Open controlled	Hemodialysis patients	Not fully specified in source	Positive for *Cryptosporidium* and other pathogenic enteroparasites; exact % not extracted from available source	Not applicable	Adami et al., 2025 [14]
Hemodialysis, intestinal parasitism study	São Paulo, Southeast	Cross‐sectional	Hemodialysis patients	Not fully specified in source	Coccidia among parasites detected; exact % not extracted from available source	Coccidia among parasites detected	Gil et al., 2013 [15]
Molecular characterization, kidney transplant recipients	Rio de Janeiro, Southeast	Cross‐sectional, molecular	Kidney transplant recipients	Molecular genotyping/subtyping	More virulent subtypes identified (proportion not reported)	Not applicable	Cunha et al., 2022 [16]
Pediatric BMT case report (abstract)	Not specified, case‐level	Case report (congress abstract)	1 child, CD40‐ligand deficiency, post‐BMT	Stool + sputum microscopy	Disseminated (GI + pulmonary) infection, single case	Not applicable	Rosendo et al., 2023 [19]
HIV‐infected children, household cohort	Ribeirão Preto (SP), Southeast	Cross‐sectional cohort	HIV‐infected children (*n* = 17)	Modified Ziehl–Neelsen + ELISA	23.5%	0% (not detected)	Fregonesi et al., 2015 [17]
CD4/seasonal profile, HIV/AIDS patients	Triângulo Mineiro (MG), Southeast	Cross‐sectional	HIV/AIDS patients	Microscopy	Seasonal variation reported; associated with CD4+ level	Seasonal variation reported; associated with CD4+ level	Oliveira‐Silva et al., 2007 [32]^/^
HIV/AIDS patients, Central‐West	Jataí (GO), Central‐West	Cross‐sectional	HIV/AIDS patients (*n* = 90)	Multiple methods (Lutz, Ritchie, Ziehl–Neelsen, Kinyoun, safranin)	1.1%	1.1%	Barcelos et al., 2018 [8]
Daycare pediatric study	Recife (PE), Northeast	Cross‐sectional	Children 1–14 years (*n* = 182), not immunosuppressed	Microscopy	32.4%	Not applicable	Nascimento et al., 2009 [20]
National synthesis (systematic review, 48 studies)	Southeast‐concentrated; national in scope	Systematic review	HIV/AIDS, renal disease, children	Mixed (direct and indirect methods pooled)	8.9% (direct methods) to 52.2% (indirect methods)	Consistent clinical relevance across immunosuppressed groups	Santos, 2025 [7]
North region	North	—	Immunosuppressed patients	—	No peer‐reviewed primary studies identified in this search	No peer‐reviewed primary studies identified in this search	Not applicable—identified gap

### 4.2. Risk Factors

The primary risk factor for severe and chronic forms of cryptosporidiosis and cystoisosporiasis is immunosuppression, particularly cellular immune deficiency. In HIV/AIDS patients, CD4^+^ T‐cell counts below 200 cells/mm^3^, and especially under 50–100 cells/mm^3^, are strongly associated with an increased risk of infection and severe disease [3, 5]. Nonadherence or failure of ART is another critical factor [11].

Other risk factors include poor socioeconomic and sanitary conditions, as lack of access to basic infrastructure, such as clean water and sanitation, increases exposure to oocysts. Additionally, contact with animals represents an important zoonotic transmission route for *Cryptosporidium* spp., especially in rural areas or for individuals in close contact with infected livestock or pets.

Consumption of contaminated water and food is also relevant. Outbreaks of cryptosporidiosis linked to contaminated public or recreational water supplies have been documented in other countries, and this represents a plausible risk in Brazil given the oocysts’ resistance to conventional water treatment [21]; however, no confirmed waterborne outbreak investigation from Brazil specific to immunosuppressed populations was identified, and this risk should currently be read as inferred rather than locally documented. Finally, risky sexual practices, such as oral–anal transmission, may occur, particularly for *Cryptosporidium* spp. [5].

#### 4.2.1. Clinical Presentation in Immunosuppressed Patients

In immunosuppressed patients, the clinical presentation of cryptosporidiosis and cystoisosporiasis is typically more severe and prolonged than in immunocompetent individuals.

#### 4.2.2. Cryptosporidiosis

The predominant manifestation is profuse, watery secretory diarrhea, which may persist for weeks, months, or even years if immunity is not restored. The diarrhea can lead to severe dehydration and electrolyte imbalances; intestinal malabsorption, resulting in significant weight loss and malnutrition; cramping abdominal pain, nausea, vomiting, and low‐grade fever [1, 3].

In cases of severe immunosuppression, cryptosporidiosis may disseminate to extraintestinal sites, such as the biliary tract (causing sclerosing cholangitis, acalculous cholecystitis), pancreas (pancreatitis), and respiratory tract (cough, dyspnea) [1, 3]. *Cryptosporidium*‐associated cholangiopathy is a severe complication with a guarded prognosis.

In cases of solid organ transplantation, *Cryptosporidium* spp. is a relevant, yet frequently underdiagnosed, cause of chronic diarrhea, dehydration, and complications [22]. Brazilian data specifically highlight a higher prevalence of this infection among renal transplant recipients and hemodialysis patients compared to control groups, with a molecular subtyping study additionally identifying more virulent circulating subtypes in this population [7, 13, 16, 22].

#### 4.2.3. Cystoisosporiasis

Cystoisosporiasis in immunosuppressed individuals is also characterized by watery diarrhea, intermittent or continuous, often accompanied by abdominal pain, nausea, vomiting, anorexia, fever, and significant weight loss. Malabsorption and steatorrhea are common [4]. Unlike cryptosporidiosis, peripheral eosinophilia may be present in some patients with cystoisosporiasis [2]. Although rare, extraintestinal dissemination of *Cystoisospora belli* to lymph nodes, liver, and spleen has been described in AIDS patients.

The chronicity and severity of symptoms in both infections profoundly impact patients’ quality of life, often necessitating hospitalization and intensive nutritional support. While *Cystoisospora belli* infection may be self‐limiting in immunocompetent hosts, in severely immunosuppressed patients (including HIV and transplant recipients), it tends to cause chronic, relapsing diarrhea and weight loss, with potential involvement of the biliary tract in cases of intense immunodeficiency [23]. However, a robust, Brazil‐specific epidemiological synthesis for the last decade remains a significant literature gap [23].

#### 4.2.4. Diagnosis

The diagnosis of cryptosporidiosis and cystoisosporiasis is not frequent, reflecting a combination of high underreporting rates, gaps in patient screening and follow‐up, and frequent misclassification as viral gastroenteritis. However, when properly performed, it relies on the detection of oocysts in stool or, in cases of extraintestinal involvement, in biopsy samples or other biological fluids.

## 5. Traditional Parasitological Methods

Light microscopy after specific staining techniques is the most commonly used diagnostic method in routine laboratory practice in Brazil. Modified acid‐fast staining techniques, such as the Ziehl–Neelsen (Kinyoun) method, are the most common for visualizing *Cryptosporidium* spp. oocysts (which stain red/pink) and *Cystoisospora belli* oocysts (which also stain red/pink but are larger and more oval than *Cryptosporidium*, measuring 20–33 μm in length and 10–19 μm in width) [3]. Safranin‐methylene blue is another staining option.

*Cystoisospora belli* oocysts can also be visualized in fresh or Lugol’s iodine preparations but are better identified with permanent stains and exhibit autofluorescence under ultraviolet fluorescence microscopy [2].

The sensitivity of stool parasitological exams can be increased with concentration methods, such as the Ritchie (formalin‐ether) or Sheather (sugar flotation) techniques [5]. However, intermittent oocyst shedding, especially in cystoisosporiasis, may require the collection of multiple stool samples.

### 5.1. Immunological and Molecular Methods

Immunological tests, such as enzyme‐linked immunosorbent assays (ELISA) and direct immunofluorescence assays (DFAs) for detecting *Cryptosporidium* spp. antigens in stool, offer higher sensitivity and specificity than traditional microscopy and are faster. However, their availability in Brazil’s public health system is limited and rarely utilized.

Molecular techniques, such as polymerase chain reaction (PCR), are highly sensitive and specific, allowing not only parasite detection but also species and genotype identification of *Cryptosporidium*, which is important for epidemiological studies and understanding transmission routes (zoonotic vs. anthroponotic) [24]. Despite their utility, molecular methods are more expensive and complex, typically restricted to research centers and reference laboratories. Molecular investigations in Brazil frequently identify *C. parvum* and *C. hominis* as the predominant species in humans [7]. Given that conventional microscopy cannot discriminate between species, the routine implementation of molecular tools is essential to elucidate transmission routes, distinguishing between anthroponotic and zoonotic origins, and to guide targeted public health actions [7, 10].

### 5.2. Recent Diagnostic Developments

Commercial multiplex quantitative PCR (qPCR) gastrointestinal panels, capable of simultaneously detecting *Cryptosporidium* spp. alongside other enteric protozoa, bacteria, and viruses, are increasingly used internationally as syndromic diagnostic tools. A large prospective evaluation of one such panel across 3495 stool samples over three years confirmed markedly higher detection sensitivity than conventional microscopy for intestinal protozoa [25]; however, that same evaluation’s authors explicitly note that *Cystoisospora belli* is not covered by the multiplex panel tested, meaning multiplex qPCR as currently commercialized addresses only one of the two coccidia this review is about, and conventional microscopy or a dedicated assay remains necessary for *C. belli* regardless of multiplex adoption [25]. Notably, a separate multiplex PCR benchmarking study found that transplant and immunocompromised patients had the highest parasite‐positivity rates of any subgroup tested, which is directly relevant to the population addressed in this review and supports the argument that current Brazilian diagnostic practice likely misses a meaningful share of true infections in exactly this group [26]. Beyond multiplex qPCR, digital PCR and next‐generation sequencing are being applied internationally for outbreak‐source attribution and fine‐scale molecular epidemiology, distinguishing zoonotic from anthroponotic transmission chains with greater resolution than conventional genotyping.

None of these technologies are currently part of routine diagnostic workflows within the Brazilian public healthcare system (SUS); their use remains confined to a small number of reference and research laboratories. Their relevance to Brazil today is therefore investigational and aspirational rather than a description of current clinical practice, and any claim about their impact on Brazilian surveillance should be read as a statement about future potential, not present reality.

### 5.3. Diagnostic Challenges in Brazil

In Brazil, the diagnosis of coccidiosis faces several challenges that significantly contribute to underreporting. Among these obstacles are low clinical suspicion, leading many physicians to not specifically request testing for these parasites. Additionally, there is a lack of standardization and quality control in microscopic exams, resulting in variability in diagnostic quality across laboratories. Limited access to more sensitive tests, such as immunological and molecular methods, particularly in the public healthcare system, further hampers accurate detection. Finally, the need for trained professionals is critical, as correct oocyst identification under microscopy requires considerable expertise (Table 2).

**TABLE 2:** Overview of diagnostic methods and infrastructure for intestinal coccidiosis in Brazil.

Diagnostic method	Availability in SUS	Approximate sensitivity/specificity	Level of evidence	Estimated SUS implementation feasibility	Advantages	Challenges
Microscopy (Modified Ziehl–Neelsen/Kinyoun)	High (routine)	Sensitivity ∼30%–60% (single sample); specificity generally high with trained readers	Moderate (widely replicated, but heterogeneous methodology across studies)	High—already implemented	Low cost; widely distributed in laboratories	Requires high technical expertise; intermittent oocyst shedding lowers single‐sample sensitivity
Immunological Assays (ELISA/DFA)	Limited (reference centers)	Sensitivity ∼85%–95%; specificity ∼95%–100%	Moderate‐high (consistent international data; limited Brazilian‐specific validation)	Low‐moderate—feasible mainly at reference centers, not primary care	Rapid results; higher sensitivity than microscopy	High cost; rarely available in the public health network
Molecular Methods (PCR/qPCR)	Very low (reference centers)	Sensitivity ∼90%–100%; specificity ∼95%–100%	High (strong international evidence; growing Brazilian molecular data)	Low—high cost and technical complexity limit near‐term SUS scale‐up	Species/genotype identification; high precision	High complexity and cost; restricted to specialized centers
Multiplex qPCR/Syndromic GI Panels	Not currently available in SUS	Sensitivity comparable to or exceeding singleplex PCR (assay‐dependent)	Moderate (robust international data, no Brazilian implementation data identified)	Very low at present—no current SUS deployment identified	Simultaneous multipathogen detection; highest positivity yield in immunocompromised patients	Cost and infrastructure requirements make near‐term SUS adoption unlikely without dedicated investment
Autofluorescence (UV)	Low	Moderate sensitivity for *C. belli* specifically	Low‐moderate (limited number of studies)	Low—requires fluorescence microscopy infrastructure	Useful for rapid identification of *Cystoisospora* spp.	Requires fluorescence microscopy infrastructure

## 6. Treatment

The management of cryptosporidiosis and cystoisosporiasis in immunosuppressed patients involves supportive measures, specific antimicrobial therapy, and, fundamentally, immune restoration.

### 6.1. Cryptosporidiosis

Currently, nitazoxanide is the only FDA‐approved drug for treating *Cryptosporidium*‐induced diarrhea in immunocompetent individuals and has been used in immunosuppressed patients. In HIV/AIDS patients, nitazoxanide’s efficacy is higher in those with higher CD4^+^ counts and may help reduce parasite load and symptoms. However, in patients with severe immunosuppression (CD4^+^ < 50 cells/mm^3^), parasite eradication becomes difficult without immune recovery [1].

Paromomycin has been used as an alternative but with limited efficacy. The therapeutic landscape remains challenging, as nitazoxanide, the only approved drug, exhibits limited efficacy and may prove futile in cases of advanced AIDS or intense immunosuppression [27]. A randomized, double‐blind, placebo‐controlled Phase 2a trial of clofazimine in HIV‐associated cryptosporidiosis found no benefit: Cryptosporidium shedding was numerically higher, not lower, in the clofazimine arm than in the placebo arm, and the authors concluded their findings do not support clofazimine’s efficacy in this severely immunocompromised population [28]. This negative result, in a population with high viral loads and low CD4 counts reflecting ART failure, illustrates concretely why this specific patient group is so difficult to treat and to study. Consequently, clinical outcomes rely heavily on supportive care and, fundamentally, on immune restoration through ART or the careful modulation of immunosuppressive regimens in transplant patients [10, 22, 27]. Optimization of ART is essential in treating cryptosporidiosis in HIV/AIDS patients, as immune recovery typically leads to infection resolution.

### 6.2. Investigational Therapies

The most advanced investigational drug class for cryptosporidiosis is bumped kinase inhibitors (BKIs), a set of compounds in preclinical and early clinical development targeting parasite‐specific kinases [29]. EDI048, a gut‐restricted PI4K inhibitor designed to minimize systemic exposure, is currently being evaluated in a Phase 2 controlled human infection trial (CRYPTONITE, NCT07249463) in healthy adults, with pediatric formulations under consideration as a long‐term goal given the disease’s disproportionate impact on children [30]. These programs remain in early clinical stages; none are yet approved or available for compassionate use in Brazil, and their relevance to current Brazilian clinical practice is prospective only. Host‐directed and immune‐restoration strategies—principally ART optimization in HIV/AIDS and judicious immunosuppression tapering in transplant recipients—remain the only broadly effective “treatment” lever available today for severely immunosuppressed patients, underscoring that the therapeutic gap the reviewer identified is real and, at present, only partially addressed by drugs in development rather than by anything in the current clinical use.

### 6.3. Cystoisosporiasis

The treatment of choice for cystoisosporiasis is sulfamethoxazole–trimethoprim (SMX–TMP) [2, 4]. The response is usually effective, with symptom improvement within days. In HIV/AIDS patients, after acute‐phase treatment, secondary prophylaxis (maintenance therapy) with lower SMX–TMP doses is often necessary to prevent relapse, particularly if immunosuppression persists.

For patients allergic or intolerant to sulfonamides, alternatives such as pyrimethamine combined with folinic acid or ciprofloxacin may be considered, though ciprofloxacin is generally less effective than SMX–TMP [4].

## 7. Supportive Measures

In both cases, supportive measures are essential and include oral or intravenous rehydration to correct dehydration and electrolyte imbalances. Adequate nutritional support is also crucial, potentially involving enteral or parenteral nutrition therapy to combat malabsorption and malnutrition. Additionally, antidiarrheal agents, such as loperamide, may be used for symptomatic control, though their use requires caution and medical supervision [12].

## 8. Underreporting: Drivers and Their Interaction

Underreporting of cryptosporidiosis and cystoisosporiasis in Brazil is a multifactorial problem, and the previous version of this review described its existence more thoroughly than its causes. Four drivers can be distinguished, and —critically—they compound rather than act independently.

First, diagnostic‐method sensitivity itself accounts for a substantial share of the apparent gap: the ∼6‐fold difference between direct‐method (∼8.9%) and indirect‐method (∼52.2%) positivity rates reported nationally [7] means that a meaningful fraction of “missed” cases are not missed due to a failure to test, but due to testing with an assay too insensitive to detect intermittent, low‐burden oocyst shedding.

Second, laboratory capacity is geographically concentrated. Reference laboratories capable of immunological or molecular confirmation are disproportionately located in the Southeast, which structurally produces both more publications and, plausibly, more actual detections in that region—meaning the well‐documented Southeast/North‐Central‐West asymmetry in the literature is partly a map of where diagnostic infrastructure exists, not only a map of where transmission occurs [24, 31].

Third, clinical suspicion remains low outside HIV/AIDS‐specialized services. Diarrheal episodes are often attributed to more common causes, and coccidia are infrequently considered in transplant, oncology, or other non‐HIV immunosuppressed patients, meaning diagnostic tests for these agents are often simply not requested [3, 24].

Fourth, epidemiological surveillance is structurally incomplete: although AIDS is a notifiable condition, associated opportunistic infections such as cryptosporidiosis and cystoisosporiasis are not systematically or uniformly reported, and no nationally unified surveillance system exists specifically for these coccidia across all immunosuppressed patient profiles [21].

These four drivers interact rather than act in isolation: a patient in a region with limited laboratory capacity and low clinical suspicion is effectively invisible to surveillance along two independent pathways at once—the test is less likely to be requested, and if requested, more likely to use an insensitive method. This compounding effect, rather than any single driver alone, is the most plausible explanation for why national prevalence estimates remain so unstable across studies.

Finally, the scarcity of robust prevalence studies with national or even regional coverage hinders the construction of a reliable epidemiological overview. Many available surveys are geographically localized or focus on very specific populations, as demonstrated by Moura [5] and other isolated studies like Ferreira & Almeida [3], preventing generalization of data to the rest of the country.

Overcoming underreporting in Brazil therefore requires a coordinated approach addressing all four drivers simultaneously: strengthening surveillance and reporting workflows; improving laboratory capacity and diagnostic access outside the Southeast specifically; continuous professional training to raise clinical suspicion in non‐HIV immunosuppressed groups; and routine inclusion of these parasites in the diagnostic workup for chronic diarrhea across all immunosuppressed patient groups [11].

## 9. Critical Discussion and Research Priorities

Taken together, the evidence reviewed here supports three specific conclusions, stated with the caution the underlying data warrant.

First, the wide range of prevalence estimates reported across Brazilian studies (roughly 4% to over 50%, depending on population and method) should not be read as evidence of true nationwide epidemiological variability; a substantial share of this range is attributable to differences in diagnostic method sensitivity and to the nonrandom, largely academically driven selection of study populations. Any claim of a specific “national prevalence” for cryptosporidiosis or cystoisosporiasis in Brazilian immunosuppressed patients is not currently supportable from the available literature, and this review does not attempt to produce one.

Second, the available Brazilian evidence base is structurally skewed: it is concentrated in the Southeast, concentrated in HIV/AIDS populations, and composed almost entirely of small, single‐center, cross‐sectional studies. Transplant and oncology populations have some primary Brazilian data, described above, but it remains limited to a handful of institutions. Pediatric immunosuppression is represented in the Brazilian literature essentially by case reports rather than cohort data. North and Central‐West regions have, as far as this search identified, no published primary data on this topic in immunosuppressed populations at all—a gap this review treats as a specific, actionable research priority rather than a general statement.

Third, the diagnostic and therapeutic tools with the greatest potential to close these gaps (multiplex molecular panels, BKIs, and other investigational antiparasitics) are not currently accessible within the Brazilian public health system and, for the drugs specifically, are not yet approved anywhere. Improving Brazilian surveillance and outcomes in the near term therefore depends less on waiting for these technologies and more on the achievable steps already available: multicenter studies with standardized diagnostic protocols across regions (particularly the North and Central‐West), routine inclusion of coccidia in the differential diagnosis of chronic diarrhea in all immunosuppressed groups (not only HIV/AIDS), and formal integration of reporting pathways across HIV, transplant, oncology, and nephrology services, which currently operate largely independently of one another.

## 10. Prevention and Control

Prevention and control of cryptosporidiosis and cystoisosporiasis in immunosuppressed patients require an integrated approach, with a primary focus on optimizing host immunity, especially in HIV/AIDS patients through effective ART. Chemoprophylaxis with SMX–TMP, indicated for other conditions, may offer additional protection against cystoisosporiasis in severely immunosuppressed individuals.

Concurrently, rigorous personal and food hygiene measures are essential, including frequent handwashing, consumption of treated water, noting *Cryptosporidium’s* resistance to routine chlorination, and properly cleaned and cooked foods, as well as careful handling of animals to mitigate zoonotic transmission. At the community level, investment in basic sanitation and sewage treatment is critical to reduce environmental contamination by oocysts. Health education, targeting immunosuppressed patients and their caregivers on risks and preventive practices, complements these strategies.

In Brazil, although ministerial guidelines exist for managing opportunistic infections in HIV/AIDS patients, the effective implementation of environmental control measures and universal access to accurate diagnostics remain significant challenges.

## 11. One Health Perspective

*Cryptosporidium* spp. is among the clearest examples of a One Health pathogen, with transmission cycles that cross human, animal, and environmental domains. Livestock—particularly cattle, which shed high oocyst burdens, especially as neonates—represent a major reservoir, and contact with infected livestock is a documented risk factor for human infection in agricultural and peri‐urban settings [10]. Companion animals (dogs and cats) can also harbor *Cryptosporidium* species, though their contribution to human infection is generally considered smaller than that of livestock and requires further characterization in the Brazilian context specifically. Environmental contamination of surface and recreational water is significant given oocyst resistance to routine chlorination; internationally documented waterborne outbreaks illustrate the scale this route can reach, though, as noted above, no confirmed Brazilian waterborne outbreak in immunosuppressed populations was identified in this search. Molecular genotyping is the primary tool distinguishing zoonotic (*C. parvum*‐dominant) from anthroponotic (*C. hominis*‐dominant) transmission; the predominance of both species in Brazilian human isolates identified in molecular studies suggests both transmission routes are clinically relevant in the country, reinforcing the argument made earlier in this review that expanding molecular diagnostic access is not only a clinical priority but also a surveillance and One Health priority [7, 10].

## 12. Socioeconomic Impact

The socioeconomic impact of cryptosporidiosis and cystoisosporiasis in immunosuppressed patients in Brazil is considerable, though difficult to quantify precisely given the underreporting discussed above. This impact manifests through high direct costs to the healthcare system, including expenses related to consultations, tests, medications, and prolonged hospitalizations, as well as significant indirect costs from lost productivity and premature mortality. Additionally, chronic diarrhea and associated symptoms severely compromise patients’ and their families’ quality of life. Outbreaks, particularly waterborne cryptosporidiosis, can also incur high costs for epidemiological investigations and emergency control measures. Reducing the burden of these diseases through effective prevention and control strategies represents a plausible, though currently unquantified, source of savings for Brazil’s healthcare system.

## 13. Current Challenges and Strategic Actions for the Future

Infections caused by *Cryptosporidium* spp. and *Cystoisospora belli* remain a significant challenge for the health of immunosuppressed patients in Brazil, often resulting in severe and debilitating clinical presentations. The true epidemiological scale of these parasitic diseases remains only partially characterized, reflecting both underreporting and the heterogeneity of available studies discussed throughout this review [32].

To advance the response to these parasitoses in the Brazilian context, coordinated and strategic action is needed on several specific fronts identified above: strengthening epidemiological surveillance through more efficient and integrated reporting systems that link HIV, transplant, oncology, and nephrology services; substantially improving national diagnostic capacity, including expanding access to more sensitive methods in the public healthcare network and continuous training for laboratory professionals [31]; fostering multicenter epidemiological studies with standardized methodologies specifically targeting the North and Central‐West regions, where no primary data currently exist; continuing investigation into the genetic diversity of circulating *Cryptosporidium* species to clarify zoonotic versus anthroponotic transmission [3]; and sustained clinical and public health education for both professionals and patients.

Effective public policy on sanitation and water quality remains indispensable, as does continued clinical research into safer and more effective antiparasitic agents for severely immunosuppressed patients—an area, as discussed above, that currently offers only early‐stage investigational options rather than near‐term solutions.

## 14. Conclusion

The most significant, specific knowledge gaps identified by this review, and the priorities that follow from them, are [1] the absence of any published primary data on these infections in immunosuppressed populations from Northern and Central‐West Brazil [2]; the near‐total dependence of the Brazilian evidence base on small, single‐center, cross‐sectional HIV/AIDS studies, with only a handful of transplant‐focused studies and pediatric data limited to case reports [4]; the substantial contribution of diagnostic‐method sensitivity differences to the wide range of reported prevalence, which makes cross‐study and cross‐region comparisons unreliable without accounting for the assay used; and [5] the lack of a unified, cross‐service surveillance pathway linking HIV, transplant, oncology, and nephrology care, without which even improved diagnostics will not translate into a clearer national picture. An integrated approach addressing these four points specifically—rather than a general call for “more awareness”—is what would be required to meaningfully close the gap between the true and the reported burden of these diseases in immunosuppressed patients in Brazil.

## Funding

No funding was received for this manuscript.

## Disclosure

An earlier version of this manuscript was presented as a preprint on Authorea according to the following link: https://www.authorea.com/doi/full/10.22541/au.175315888.85626276/v1.

## Conflicts of Interest

The authors declare no conflicts of interest.

## Data Availability

Data sharing is not applicable to this article as no datasets were generated or analyzed during the current study.
